# Title NMR-based metabolic profiling provides diagnostic and prognostic information in critically ill children with suspected infection

**DOI:** 10.1038/s41598-020-77319-0

**Published:** 2020-11-19

**Authors:** Arturas Grauslys, Marie M. Phelan, Caroline Broughton, Paul B. Baines, Rebecca Jennings, Sarah Siner, Stephane C. Paulus, Enitan D. Carrol

**Affiliations:** 1 University of Liverpool Institute of Infection and Global Health, Ronald Ross Building, 8 West Derby Street, Liverpool, L69 7BE UK; 2 Department of Infectious Diseases, Alder Hey Children’s NHS Foundation Trust, Eaton Road, Liverpool, L12 2AP UK; 3 Liverpool Health Partners, 1st Floor, Liverpool Science Park, 131 Mount Pleasant, Liverpool, L3 5TF UK; 4 University of Liverpool Institute of Integrative Biology, Biosciences Building, Crown Street, Liverpool, L69 7ZB UK; 5 Department of Critical Care, Alder Hey Children’s NHS Foundation Trust, Eaton Road, Liverpool, L12 2AP UK; 6 Medicine, Ethics, Society and History, University of Birmingham, Birmingham, UK; 7 Clinical Research Division, Alder Hey Children’s NHS Foundation Trust, Liverpool, UK; 8 Department of Infectious Diseases, Oxford University Hospital, Children’s Hospital, John Radcliffe Hospital, Oxford, OX3 9DU UK

**Keywords:** Biochemistry, Computational biology and bioinformatics, Microbiology, Biomarkers, Health care, Medical research

## Abstract

Sepsis, defined as life-threatening organ dysfunction caused by infection is difficult to distinguish clinically from infection or post-operative inflammation. We hypothesized that in a heterogeneous group of critically ill children, there would be different metabolic profiles between post-operative inflammation, bacterial and viral infection and infection with or without organ dysfunction. 1D ^1^H nuclear magnetic resonance spectra were acquired in plasma samples from critically ill children. We included children with bacterial (n = 25) and viral infection (n = 30) and controls (n = 58) (elective cardiac surgery without infection). Principal component analysis was used for data exploration and partial least squares discriminant analysis models for the differences between groups. Area under receiver operating characteristic curve (AUC) values were used to evaluate the models. Univariate analysis demonstrated differences between controls and bacterial and viral infection. There was excellent discrimination between bacterial and control (AUC = 0.94), and viral and control (AUC = 0.83), with slightly more modest discrimination between bacterial and viral (AUC = 0.78). There was modest discrimination (AUC = 0.73) between sepsis with organ dysfunction and infection with no organ dysfunction. In critically ill children, NMR metabolomics differentiates well between those with a post-operative inflammation but no infection, and those with infection (bacterial and viral), and between sepsis and infection.

## Introduction

Sepsis, defined as life-threatening organ dysfunction caused by a dysregulated host response to infection, is a medical emergency^1^. Diagnosis of sepsis can be difficult in the early stages, with non-specific symptoms. The host response to sepsis is an inflammatory cascade, with over-activation of the immune system, which without urgent treatment can rapidly lead to death. Newer definitions of sepsis have been developed for adults^1^, but not children, and older definitions^2^ fail to adequately characterise clinical presentations of sepsis. There is significant biological and clinical heterogeneity in individual patients with a clinical diagnosis of “sepsis”.

There are several hundred biomarkers of infection that have been studied, but none has been established as a sensitive and specific test for the diagnosis of sepsis. Blood cultures take 24–36 h for initial results, but polymerase chain reaction (PCR) of blood or cerebrospinal fluid (CSF) can diagnose infection more rapidly than standard culture techniques. Ascertainment of bacterial aetiology is based on detection by culture, PCR or antigen in a sterile site, such as blood, cerebrospinal fluid, urine or intra-operative specimens. Viral aetiology is ascertained by viral PCR of respiratory specimens, blood, urine, CSF or other sterile site fluid. These microbiological investigations is may be augmented by laboratory tests (biochemical and haematological) and imaging studies such as chest X-ray, CT or MRI scan.

Molecular signatures from transcriptomic, metabolomic or proteomic analysis could potentially lead to more rapid diagnosis and better characterization of specific sub-populations subsets for targeted therapies. Metabolomics has been applied to blood samples of patients with sepsis to attempt to determine the disease severity^3,4^, or patient outcome^5–7^. Sepsis results in alterations in concentrations of chemicals and small molecules in the blood, which can be measured and compared between different phenotypes or subtypes of sepsis, and in patients with different outcomes.

In this study we aimed to determine if, in critically ill children, plasma metabolomic profiling could distinguish between (a) infection versus post-operative inflammation without infection, (b) bacterial versus viral aetiology, (c) sepsis (infection with organ dysfunction) versus infection without organ dysfunction.

## Results

### Patient cohorts

Consecutive children admitted to a large tertiary centre paediatric intensive care unit (PICU), were recruited and categorised into those with bacterial or viral infection and controls (post-operative cardiac surgery with no infection). Characteristics of the cohort are summarised in Table 1 and Supplementary Fig. S1. Inclusion criteria from the patient cohort were children aged birth to 16 years, consecutively admitted to PICU between October 2010 and June 2012. Consecutive admissions were selected if there was sufficient plasma for metabolomic analysis, and if there was a clean phenotype of defined criteria. The groups were as closely age and sex matched as possible.

**Table 1:** Key characteristics of patient cohort.

	Bacterial infection (N = 25)	Viral infection (N = 30)	Controls (N = 58)
Age, median (range), months	28.4 (8.2–103.4)	9.3 (0.8–156.6)	16.2 (0.12–197.2)
Male, No. (%)	14 (56)	19 (63)	29 (50)
MODS, No. (%)	18 (72)	7 (23)	1 (1.7)
LOS on PICU, median (range), days	6.5 (0.5–26.6)	3.9 (1.7–45.6)	1.3 (0.57–29.4)
Deaths No	1	0	1
Pathogens	Klebsiella pneumoniae (2) Methicillin Resistant Staphylococcus aureus (1) Neisseria meningitidis (11) Pseudomonas aeruginosa (2) Stenotrophomonas maltophilia (1) Streptococcus pneumoniae (4) Streptococcus pyogenes (4)	Adenovirus (2) Coronavirus (2) Herpes Simplex Virus (1) Influenza A (3) Respiratory Syncytial Virus (13) Rhinovirus ( 9) Varicella Zoster Virus ( 1) (1 patient had more than 1 virus)	

*MODS* multiple organ dysfunction patient count, *LOS* length of stay, *PICU* paediatric intensive care unit.

Diagnoses were defined according to Herberg et al.^8^ Control (C), (post-operative cardiac surgery patients with no infection), bacterial (definite bacterial (DB), children with culture or PCR-positive infection form a sterile site), viral (definite viral (DV), children with PCR, rapid antigen or immunofluorescent test-confirmed viral infection and no features or evidence of co-existing bacterial infections presenting with sepsis that was culture negative). Multiple organ dysfunction was defined as per Goldstein et al.^2^.

### Optimisation of cohort criteria to produce clean phenotypes

Furthermore, post data acquisition an outlier detection algorithm identified two outliers in the control group and one viral outlier. Closer inspection of the spectra showed that one patient’s plasma had an extremely high level of citrate which may be indicative of sample collection in a non-approved plasma collection tube [plasma should be collected for nuclear magnetic resonance (NMR) metabolomics in heparin tubes as opposed to citrate^9,10^. Outlier samples were removed from further analysis.

### Metabolite identification

NMR spectra for all cohorts showed a consistent set of metabolite signals present with multiple metabolites annotated from 1D multiplet pattern overlap. NMR spectra of extracts were divided up into individual spectral bins accounting for one or more multiplets. The number of spectral bins in each sample were 144 of which 90 (62.5%) were assigned to 34 metabolites.

Correlations between different peaks assigned to the same metabolite were used in order to remove redundant peaks. Pairwise correlations were calculated for each metabolite that exhibited multiple peaks in NMR ^1^H spectra. The peaks that had correlations greater than 0.9 were removed leaving one representative peak. These included isoleucine, citrate, adipate and ornithine. Peaks that showed lower correlations were likely a product of peak overlaps in the spectra. As such a final decision could not be objectively made about the relative abundance of the metabolite and such peaks were left in as duplicates in order to not lose information. Therefore, duplicated metabolites should be interpreted as lower confidence signals.

### Statistical analysis and differential abundance of metabolites between groups

#### Definite bacterial and definite viral versus control cohort

**Figure 1:**
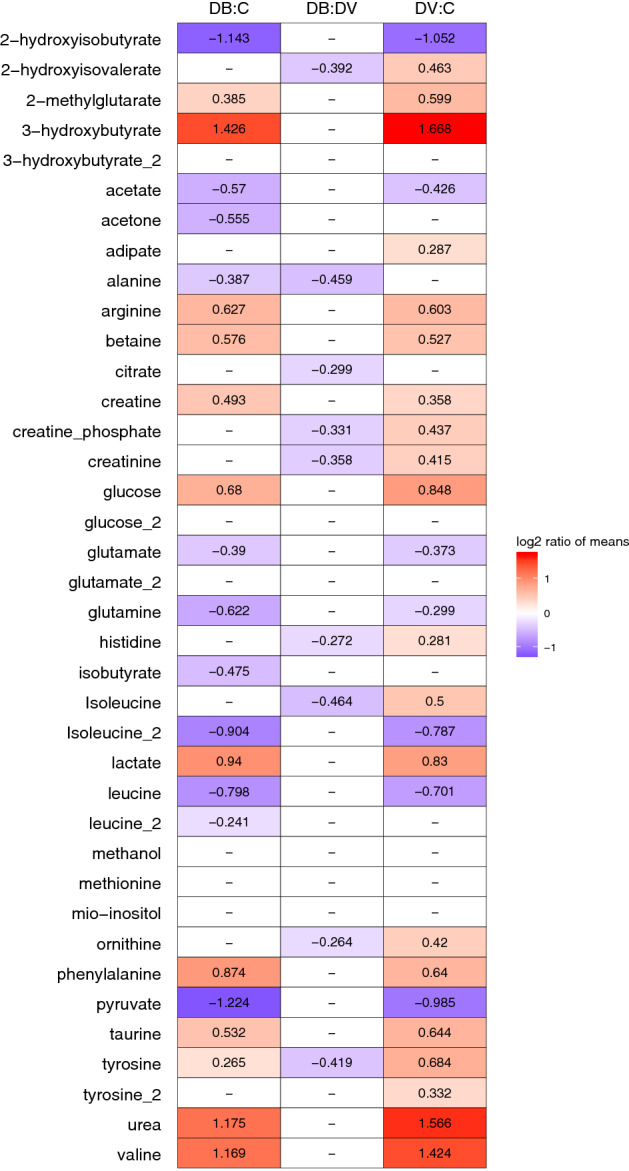
Metabolite abundance comparison. Each comparison is shown in column (*C* control, *DV* definite viral, *DB* definite bacterial). The numbers and colours denote log ratio of group means in statistically significant comparisons only (t-test, alpha = 0.05). Red means increased in DB:C or DV:C, purple lower in DB. DV relative to C or lower in DB relative to DV.

Statistical analysis of the two infected groups showed very distinct characteristics to the control population. Samples from each infected condition were compared to controls first using a t test for each metabolite signal separately, followed by multivariate analysis using PCA and sPLSDA models. The results of the univariate analysis (Fig. 1) showed that the majority of differences were present between controls and infected groups. Around two-fold or higher increase over controls was observed for 3-hydroxybutyrate, lactate, phenylalanine, urea and valine and decrease in 2-hydroxyisobutyrate, isoleucine and pyruvate. Modest, but statistically significant increase in 2-hydroxyisovalerate, adipate, creatine phosphate, creatinine and isoleucine were also observed in the comparison of viral infections to controls while not present in the bacterial samples. In turn the bacterial samples showed decreased abundance of acetone, alanine and isobutyrate that was not observed in the viral cohort. Unsupervised analysis using PCA did not reveal substantial patterns in the data (Supplementary Figs. S2, S3) with only subtle differences between groups.

**Figure 2:**
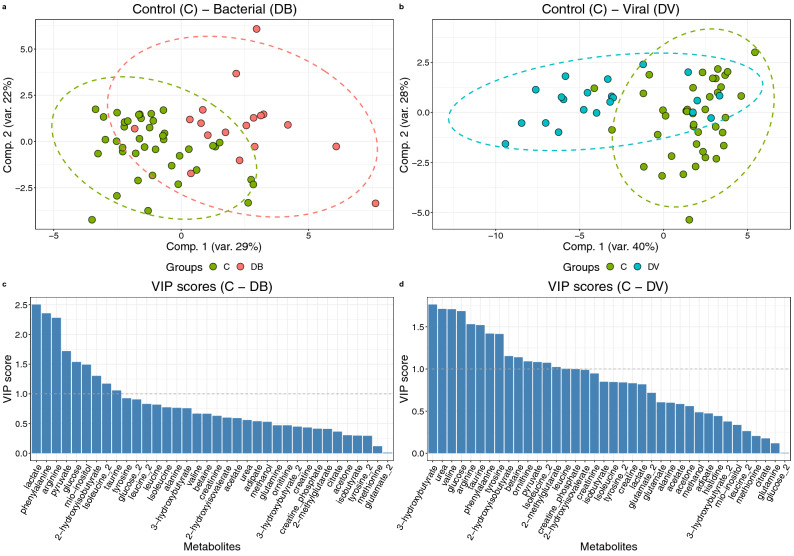
Sparse partial least squares discriminant analysis (**a**) scores plot of model including DB (N = 25) and C (N = 58); (**b**) scores plot of model including DV (30) and C (N = 58); the most contributing metabolite signals (VIP scores); (**c**) C and DB; (**d**) C and DV. Associated AUC = 0.94 in DB-C and 0.83 in DV-C. *DB*—definite bacterial, *DV* – definite viral, *C* control, *VIP* variable importance score.

Discriminant analysis via sPLS-DA distinguished either viral or bacterial infection groups from the control population (Fig. 2a,b). In the process, contributing metabolites were identified (Fig. 2c,d). The area under the ROC curves (AUC) implied that the separation achieved by the models was good to excellent with AUC values of 0.84 between DV and C, and 0.93 between DB and C (Supplementary Figs. S5A,B, S6).

Amo0ng selected metabolites arginine, glucose, isoleucine and tyrosine were highly ranked in terms of contribution in both models. In the model aimed to distinguish patients with bacterial infection from controls, myo-inositol, phenylalanine, lactate, pyruvate and 2-hydroxyisobutyrate were especially important. Conversely the model fit to distinguish viral infections from controls favoured 3-hydroxybutyrate, urea, valine 2-methylglutarate and isobutyrate.

Interestingly, most changes in metabolite abundance with respect to control patients are shared in both bacterial and viral infections (Supplementary Fig. S2). The fold changes of most measured metabolites point to similar changes in both infections with some notable exceptions including 2-hydroxyisovalerate, adipate, glutamine and histidine.

#### Bacterial versus viral infection

Similar analysis strategy was followed in the attempts to discriminate patients with bacterial and viral infection. The univariate tests showed a number of significant metabolite differences (albeit modest fold-changes) with all metabolites more abundant in samples from patients with viral infections (Fig. 1). These included 2-hydroxyisovalerate, alanine, citrate, creatine phosphate, creatinine, histidine, isoleucine, ornithine and tyrosine (Supplementary Fig. S8).

**Figure 3:**
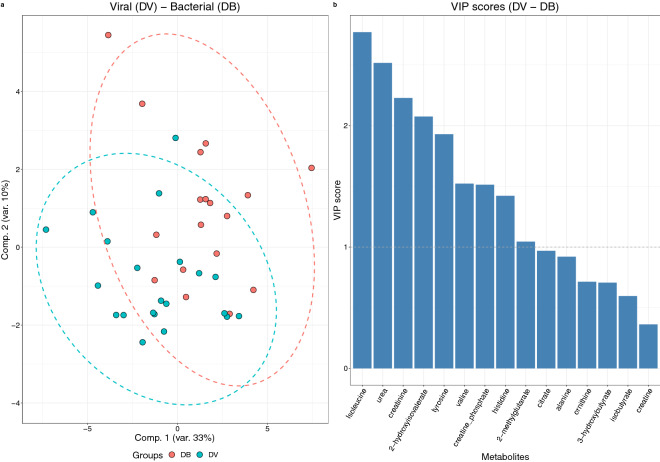
PLS-DA (**a**) scores plot of model including DB (N = 25) and DV (N = 30) samples. (**b**) Most contributing metabolite signals (VIP scores). Associated AUC = 0.78.

While PCA did not reveal clear discrimination (Supplementary Fig. S7) the sPLS-DA model showed modest discrimination between the groups (AUC = 0.78, Supplementary Figs. S6, S9) that, although unlikely to be sufficient for discriminant diagnoses, suggested biological pathways deviating between the two conditions via scrutiny of the metabolite differences. Metabolites ranked higher as discriminatory between samples from patients with bacterial and viral infections were isoleucine, urea, creatinine, 2-hydroxyisovalerate, tyrosine, valine, creatine phosphate and histidine among others (Fig. 3, Supplementary Fig. S8).

#### Metabolite association with organ dysfunction

Organ dysfunction occurs in a subset of patients with sepsis, and as such metabolic profiles have previously been associated with dysfunction^4^. Plasma short- and medium-chain acylcarnitines are associated with sepsis-related hepatobiliary and renal dysfunction, and might be markers of metabolic perturbation in liver and kidney. Acetylcarnitine is associated with organ dysfunction, systemic inflammation, and prognosis in sepsis^4^.

**Figure 4:**
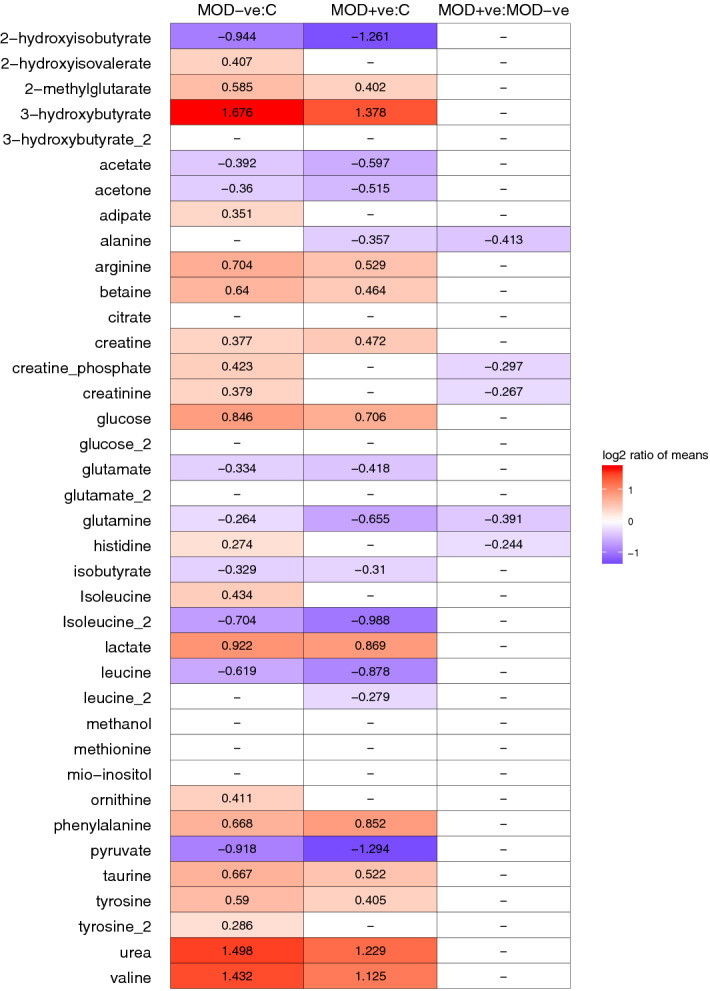
Comparison of metabolic profiles for MOD, no MOD and control cohorts. (**a**) Metabolite abundance comparison. Each comparison is shown in column (C—control, Yes—multiple organ dysfunction, No—no multiple organ dysfunction). The numbers and colours denote log ratio of group means in statistically significant comparisons only (t test, alpha = 0.05). Mean log ratios of MOD-positive and MOD negative group metabolic profiles with controls.

**Figure 5:**
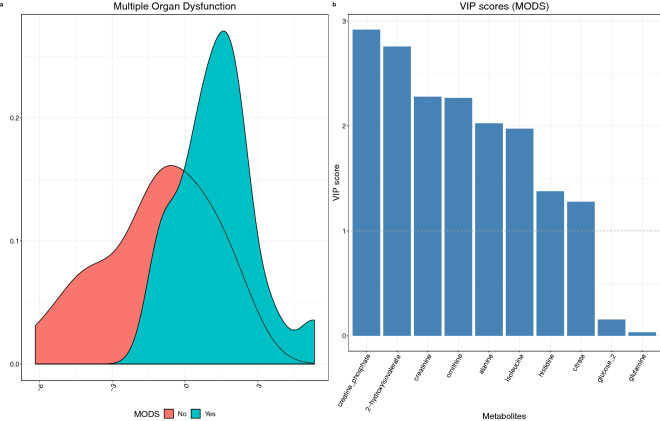
Metabolite profiles were investigated by pairwise comparisons of cohorts with and without MOD and fitting a PLS-DA model for selection of discriminating metabolites. The results show (**a**) scores plot (1 component) of model including MOD positive patients with infection (blue) (N = 25) vs MOD negative patients with infection (red) (N = 30). (**b**) Metabolite importance (VIP) plot. Associated AUC = 0.73.

Metabolite profiles were assessed with respect to multiple organ dysfunction (MOD), and it is important to note that the prevalence of MOD differed between children with bacterial and viral infections. There were 18 patients with MOD out of 25 (72%) in the cohort with bacterial infection and 7 out of 30 (23%) in the viral cohort. Metabolite profiles were again investigated by pairwise comparisons of cohorts with and without MOD to controls and each other (Fig. 4) followed by fitting a sPLS-DA model for selection of discriminating metabolites (Fig. 5).

Both children with MOD and those without MOD show similar alterations when compared to the control cohort with a few notable differences (Fig. 4). 2-Hydroxyisovalerate, adipate, creatine phosphate, creatinine, histidine, isoleucine and ornithine show mild increase in abundance compared to control in those without MOD that is not observed in samples from those with MOD, while leucine is lower in those without MOD. When compared to each other, both cohorts of patients with infection show lower levels of alanine, creatine phosphate, creatinine, glutamine and histidine in those with MOD. The differences are further illustrated in Supplementary Fig. S12.

A sPLS-DA model showed modest discrimination between the MOD-positive and MOD-negative group (AUC = 0.73, Fig. 5, Supplementary Fig. S13). The most influential metabolites highlighted by the model were 2-hydroxyisovalerate, ornithine, isoleucine, creatinine, creatine phosphate and citrate among others (Fig. 5). Notably all the metabolites were more abundant in the samples from MOD-negative patients.

#### Biological contextualisation

Previous studies on different patient cohorts (primarily in North America) have posited multiple sepsis associated metabolites (Table 2)^6,11–19^. This UK-based study which includes confirmed bacterial and viral infection concurs with some metabolite findings indicative of global consensus between populations with infection. Other metabolites that were not reported previously may be due to smaller sample size, different cohorts (adults/children, bacteraemic/non-bacteraemic, bacterial/viral) or potentially confounding factors arising from variation between different populations (genetic, environmental, severity).

**Table 2:** Metabolites reported in the literature in published studies on infection and sepsis, that were also differentially increased or decreased in our cohort.

	Metabolite ID^a^	Mickiewicz 2013^11^	Mickiewicz 2015^12^	Mickiewicz 2015^13^	Kamisoglu 2015^14^	Venet 2017^15^	Lin 2009 ^16^	Izquierdo-Garcia 2011^17^	Xu 2008 ^18^	Liu 2016 ^19^	Fanos 2014 ^20^	Our study
l-Alpha-aminobutyric acid	00452	*										
2-Hydroxybutyric acid	00008	*	+	–			+					+
2-Hydroxyisobutyric acid	00729	*										–
2-Hydroxy-3-methylbutyric acid	00407	*		-								+
2-Methylglutaric acid	00422	*										
2-Methylhippuric acid	11723									*		
Ketoleucine	00695	*		–								
3-Hydroxybutyric acid	00357	*	+									+
3-Hydroxyisovaleric acid	00754	*	–									
3-Methyl-2-oxovaleric acid	00491			–								
Acetic acid	00042	*		+			+				+	–
Acetoacetic acid	00060						+	+				
Acetone	01659	*									+	–
Adipic acid	00448	*										+
l-Alanine	00161	*		+		*	+	+				–
Oxoglutaric acid	00208									*		
l-Arginine	00517	*	–			*						+
Argininosuccinic acid	00052									*		
l-Asparagine	00168	*										
Betaine	00043	*								*		+
l-Carnitine	00062	*								*		
Choline	00097			+		*						
Citric acid	00094	*								*	+	*
Citrulline	00904									*		
Creatine	00064	*						+		*		+
Creatinine	00562	*								*		+
Dimethylamine	00087			–								
Ethanol	00108	*										
Formic acid	00142						–	–	+			
D-Glucose	00122	*	–			*					+	+
l-Glutamic acid	00148	*	–							*		–
Glutamate-Glutamine												
l-Glutamine	00641	*								*		–
Glutathione	00125					*						
Glycerol	00131	*										
Glycine	00123	*									+	
Hippuric acid	00714									*		
l-Histidine	00177	*										+
Hypoxanthine	00157	*								*		
Indolelactic acid	00671									*		
Indoxyl sulfate	00682									*		
inosine	00195					*						
Isobutyric acid	01873	*	+									–
l-Isoleucine	00172	*				*				*		*
l-Lactic acid	00190	*				*	+			*	+	–
l-Leucine	00687					*				*		–
l-Lysine	00182					*					+	
Malic acid	00744									*		
D-Mannose	00169			–								
Methanol	01875	*	–									
l-Methionine	00696	*				*						
myo-Inositol	00211	*	+									
O-Acetylcarnitine	00201	*	+									
Acetylcholine	00895			+		*						
Ornithine	00214	*								*		+
l-Phenylalanine	00159	*	+							*		+
Phosphoryl-choline	01565					*						
l-Proline	00162		+			*				*		
Propylene glycol	01881		-	-								
Pyroglutamic acid	00267	*										
Pyruvic acid	00243	*										-
l-Serine	00187	*										
Suberic acid	00893	*										
Succinic acid	00254									*		
Taurine	00251	*		+								+
l-Threonine	00167	*	-			*						
l-Tyrosine	00158	*								*		+
Urea	00294	*										+
l-Valine	00883		-			*				*		+

Metabolites levels increase or decrease with sepsis/infection as indicated by + or –, respectively. Metabolites associated with sepsis/infection but levels not specified (or differentially attributed) are indicated by *.

^†^All numbers preceded by ‘HMDB00’.

The correlations of biochemical test results present for majority of patients and selected metabolites are shown in Supplementary Table S1.

Of the metabolites listed in Table 2, only 8 were associated with infection in the Human Metabolome Database (HMDB). These metabolites were 2-aminobutyrate, glutamate, phenylalanine, pyruvate, 3-hydroxybutyrate, tyrosine, urea, and dimethylamine (DMA).

## Discussion

This study demonstrates that several metabolites can be used to distinguish between bacterial and viral infection in critically ill children. Metabolite abundance differs between bacterial, viral and control respectively with excellent discrimination between bacterial and control (AUC = 0.94) and good discrimination between viral and control (AUC = 0.83). There was modest discrimination between bacterial and viral (AUC = 0.78), which reflects the fact that many infections in children go unconfirmed, and there is no valid reference standard for studies of biomarkers in sepsis. Despite decades of research for a “gold standard” biomarker of invasive bacterial infections, only few biomarkers have been translated into routine clinical practice, with C-reactive protein (CRP) and procalcitonin being the most frequently used. Although blood culture has been considered as the “gold standard”, the process takes at least 48 h to result and is limited by false negative results. Use of CRP is hampered by a physiological 3-day increase, resulting in a low sensitivity to detect sepsis at an early stage^20^. We also demonstrated good discrimination between bacterial infection with organ dysfunction versus infection (bacterial or viral) without organ dysfunction.

A study of patients with septic shock who had NMR spectroscopy-based metabolomics profiling, reported increased concentrations of phenylalanine, myo-inositol, isobutyrate, 3-hydroxybutyrate, urea, O-acetylcarnitine, 2-hydroxybutyrate and proline, and decreased concentrations of propylene glycol, threonine, valine, arginine, glutamate, methanol and glucose^6^. This supports our findings of increased phenylalanine, 2-hydroxybutyrate and 3-hydroxybutyrate in bacterial infection. However, in our study, valine, arginine, methanol and glucose were increased in bacterial and viral infection compared to controls, and glutamate was decreased. Most of our patients did not have septic shock, which explains this difference. In a study examining patients with septic shock in intensive care, six metabolites were identified as differentiating between survivors and non-survivors. Carnitine and citrulline were decreased in septic non-survivors, betaine, valine, leucine and isoleucine were increased in septic non-survivors^18^. In our study, we also demonstrated increased betaine, valine, leucine and isoleucine in bacterial infection compared to controls. The list of metabolites differentially abundant in MOD versus controls are also all present in the comparison between definite bacterial versus control and definite viral versus control. There were 5 metabolites which were had lower levels in those with MOD compared to those without; histidine, glutamine, creatinine, creatine phosphate, alanine. In patients with sepsis, increasing phosphoserine concentrations were associated with higher severity of illness, kidney and liver dysfunction and death, and correlated with increasing cystathionine, 3-methylhistidine, histidine, hydroxyproline and tyrosine^21^. Another study of patients with septic shock, phosphatidylcholines, lysophosphatidylcholines, acylcarnitines, and sphingomyelins were decreased in non-survivors, while kynurenine and polyunsaturated diacyl-phosphatidylcholines were increased. In non-survivors, glucogenic amino acids, putrescine and spermidine, were also increased. Phosphatidylcholines, lysophosphatidylcholines were also lower in patients with renal dysfunction^22^**.** In our study, with limited sample volume available, we were unable to measure lipid metabolites beyond phosphocholine because we did not also use a mass spectrometry (MS) based technology like liquid-chromatography coupled mass spectrometry (LC–MS).

The 6 metabolites with the strongest ability to separate bacteraemic sepsis in the Emergency Department (ED) from those without sepsis were myristic acid, citric acid, isoleucine, norleucine, pyruvic acid and a phosphocholine^23^. Decreased levels of isoleucine and norleucine in bacteraemic patients is consistent with our findings of lower leucine and isoleucine in definite bacterial group compared to controls^23^. We demonstrated increased lactate in cases compared to controls.

One possible reason for the discrepancy in results between our study and previous studies, could relate to the timing of sampling. Metabolite profiles differ depending on timing of sampling. The samples in this study were taken on admission to PICU, but as the timing of onset of sepsis is unknown and there are varying lengths of time between first presentation to the ED and admission to PICU, this can result in variations in metabolic response profiles^13^. Most previous studies have compared sepsis with systemic inflammatory response syndrome or controls, or bacterial with viral, but in this study, we have compared patients with infection and MOD to those with infection without MOD. Furthermore, this study has considered the influence of MOD upon investigation of these differences no observable effects were found (Supplementary Figs. S11, S12). Of the list of metabolites associated with sepsis or bacterial infection, only 8 of them were associated with infection in the HMDB database. This reflects how little information on sepsis is included in the HMDB database. Studies such as ours are important in contributing to the publicly available data, which us why we have deposited the data to the EMBL-EBI MetaboLights database (acc. num. MTBLS563).

Our study has several strengths; we compared bacterial versus viral, as well as bacterial versus controls and viral versus controls. Our control group consisted of children admitted to critical care without infection, these children were post-operative cardiac patients who were likely to have metabolite derangements as a result of surgery and intensive care management, therefore represents a good control group. Additionally, we examined metabolite changes with organ dysfunction, which is now a key component of the new sepsis definition^1^. Previous studies, including our own^24^ have failed to show significant differences in single biomarkers between post-operative cardiac surgery patients with and without infection, and this study demonstrates that metabolic profiles are different between these groups. Limitations of our study include the fact that the number of patients with definite bacterial infection was low, and those with sepsis (infection with organ dysfunction) was even lower. This may explain why some findings from studies exclusively on children with sepsis were not replicated. Other limitations are that it is a single centre study, and there was no validation cohort, and therefore similar studies in different settings are required to determine generalisability.

Our study provides important new insights into the metabolic derangements associated with infection and organ dysfunction. The sepsis-3 definitions, developed for adult patients, have included organ dysfunction and infection for a diagnosis of sepsis, but these definitions may need to be modified for children^1^. Our data suggest that children with infection and organ dysfunction (sepsis), have a different metabolic phenotype to those with infection and no organ dysfunction. Recent studies have identified different phenotypes of adult sepsis based on host-response and organ dysfunction^25^, and in paediatric sepsis, phenotypes in whom fluid bolus with unbuffered electrolyte solutions may be harmful^26^. Defining accurate phenotypes within the clinical definition of “sepsis” has important implications for directing the optimal management strategy for each phenotype, in order to ensure the best outcomes.

In this well characterised cohort of critically ill children, metabolomic profiling demonstrated good discrimination between infection and post-operative inflammation, and between sepsis (bacterial infection with organ dysfunction) and infection without organ dysfunction.

## Methods

### Participants

Children from birth to 16 years admitted to the PICU between October 2010 and June 2012 were eligible for inclusion. Pre-term infants < 37 weeks corrected gestation, children predicted not to survive at least 28 days due to a pre-existing condition or with an existing directive to withhold life-sustaining treatment, children with end stage renal disease requiring chronic dialysis, end-stage liver disease, children admitted moribund and not expected to survive more than 24 h and non-intubated elective admissions with a predicted duration of stay less than 24 h were excluded from the study (Supplementary Fig. S1).

All methods were carried out in accordance with relevant guidelines and regulations. Written informed consent for participation in the study was obtained from parents or guardians. Children were followed up until day 28 after admission. The study was approved by the local research ethics committee (REC reference number: 10/H1014/52-NRES Committee North West).

### Sample collection

Blood samples were taken on admission to PICU in lithium heparin tubes (Sarsedt, Numbrecht Germany) at the same time as routine investigations. Samples were then frozen at – 80 °C within 2 h of collection and stored at – 80 °C until NMR analysis. Parameters of routine haematological and biochemical tests taken on admission, were also recorded (full blood count, renal function, liver function, coagulation and C-reactive protein).

### Sample groups

Sample groups for the purpose of determining a clean phenotype were defined as infants and children older than 4 weeks with an age range of 4 weeks–14.33 years of age (0.08–14.33). All eligible patients as defined by the sample group criteria were selected for the bacterial and viral infection groups. To reduce effect of age and gender the control group samples were selected to match as closely as possible with gender split in the sample groups between 36 and 50% (Table 1).

Patients were assigned to diagnostic groups using predefined criteria as per Herberg^8^. The following definitions were used:

- Definite bacterial infection: children with culture or PCR-confirmed infection from a sterile site
- Definite viral infection: children with PCR or immunofluorescent test–confirmed viral infection and no features of co-existing bacterial infection
- Sepsis: life-threatening organ dysfunction caused by a dysregulated host response to infection^1^. Organ dysfunction was defined according to Goldstein 2005 and Multiple Organ Dysfunction (MOD) defined as or two or more organ dysfunctions (respiratory, renal, neurologic, hematologic, or hepatic)^2^.
- Controls: post-operative cardiac surgery patients admitted electively to critical care, in whom no infection was diagnosed. These patients have previously been described, and had evidence of post-operative inflammation with raised Procalcitonin and C-reactive protein on post-operative day 2 and 3^24^.

### Sample preparation for NMR

100ul of serum was thawed on ice before addition of buffer to create a final sample composition of 100 mM sodium phosphate pH 7.40, 0.02% (w/v) sodium azide to a final volume of 200 μl containing 10% ^2^H_2_O. The sample was vortexed for 30 s and then centrifuged (21,500*g*, 4 °C, 5 min). 180 μl of supernatant was then transferred to a 3 mm outer diameter NMR tube using a glass Pasteur with care taken not to disturb any pelleted material.

### NMR setup acquisition and processing

1D ^1^H NMR spectra were acquired on a Bruker Avance III HD 700 spectrometer equipped with a 5 mm TCI cryoprobe and SampleJet autosampler. Spectra acquired at pH 7.4 and 37 °C using standard vendors pulse sequence with the cpmgpr1d using Carr-Purcell-Meiboom-Gill (CPMG) filters for selective observation of low molecular weight components with optimal water suppression were acquired. Spectra were acquired with 32 transients a 20 ppm spectral width, 64 K points, 9.6 ms echo time and a 3.1 s acquisition time and a 4 s interscan delay.

### Spectra processing and quality control

Automated data processing, Fourier transformation, and phasing were performed in Topspin v3.2 (Topspin 3.2.pl7 https://www.bruker.com/products/mr/nmr/software/topspin.html) software using standard Bruker routines. The spectra were referenced indirectly to TSP via the anomeric glucose doublet at 5.204 ppm and then normalised to via probabilistic quotient normalisation^27^. The spectra were then bucketed per peak into a matrix of metabolite peak intensities using custom R scripts.

### Metabolite annotation and identification

Chenomx v 8.2 (Chenomx 8.2 https://www.chenomx.com/) software was used for initial metabolite annotation followed by manual confirmation of identities using in-house standards for metabolite peaks. Identification and annotation levels are reported in the deposited data according to Metabolomics standards initiative guidelines^28–31^. Raw data, experimental parameters, annotations and identities are deposited in the open source repository Metabolights hosted by the European Bioinformatics Institute (EBI)^32^ accession number MTBLS563.

### Statistical analysis

Multivariate statistical analysis was performed using R statistical computing environment [r-project.org]^33^. Data was first normalized using probabilistic quotient normalisation to account for potential dilution effects in the samples. Principal component analysis (PCA) was then used for data exploration. An automatic outlier detection, based on Mahalanobis distance of samples in the PCA within each group, was performed. In short, the method relied on calculation of Mahalanobis distance to the centroid of each group of samples in the PC space taking the number of principal components that accounted for at least 95% of the variance. A distance cut-off was then calculated as a 99th percentile of the Chi-squared distribution with degrees of freedom set to the chosen number of principal components. Points further from the centroid than the cut-off distance were flagged as outliers. Three samples were removed from the data as outliers. The samples were split into three groups based on the criteria described by Herberg^8^—control, definite bacterial infection, definite viral infection. The samples from patients with unknown or unclear diagnosis were excluded from the analysis. The relative metabolite concentrations were compared between groups using a Student’s t test with p value correction for multiple comparisons using Benjamini–Hochberg method.

In order to assess the differences between diagnosis groups sparse partial least squares discriminant analysis (sPLS-DA) models were built. PLS-DA is a multivariate method that finds a low-dimensional representation of data maximising the correlation with the grouping (e.g. diagnosis classes). Sparse PLS-DA includes implicit variable selection based on Lasso regression^34^ which allows automated selection of most contributing variables. In order to avoid overfitting fivefold cross-validation was used for selecting the number of PLS-DA components. Models were assessed using receiver operating characteristic (ROC) curves using a hold-out dataset that was not used in the fitting of the models. Hold out datasets used for each model comprised of randomly selected subset of 30% of the patients, keeping the proportions of the groups (control-bacterial, control-viral etc.) similar to remaining 70%. Models were then fit to the remaining 70% of the data and tested on the hold out dataset to assess the model performance without bias. ROC curves were calculated from the predicted values. sPLS-DA scores plots as well as ROC curves and area under ROC curve (AUROC) values were assessed in order to evaluate the models. The modelling was performed using MixOmics^35^ package in R statistical computing environment.

### Ethics

Written informed consent for participation in the study was obtained from parents or guardians. Children were followed up until day 28 after admission. The study was approved by the local research ethics committee (REC reference number: 10/H1014/52).

## Supplementary information

Supplementary Information.

## Data Availability

Raw data, experimental parameters, annotations and identities are deposited in the open source repository Metabolights hosted by the European Bioinformatics Institute (EBI)^32^ accession number MTBLS563.
