# Supplementary material for: Title NMR-based metabolic profiling provides diagnostic and prognostic information in critically ill children with suspected infection
Source: Sci Rep. 2020 Nov 19;10:20198. doi: 10.1038/s41598-020-77319-0 (PMC7677384; doi:10.1038/s41598-020-77319-0)
Supplement: Supplementary file 1 — Supplementary Information. [file 41598_2020_77319_MOESM1_ESM.docx]

**Title NMR-based metabolic profiling provides diagnostic and prognostic information in critically ill children with suspected infection.**

**Running title: Metabolomic profiling of infection in critically ill children**

**Authors** Arturas Grauslys^4^ , Marie M Phelan ^4^, Caroline Broughton*,* ^,1^ Paul B Baines^5,6^, Rebecca Jennings ^7^, Sarah Siner S^7^ , Stephane C Paulus^1,,8^ Enitan D Carrol*^1,2, 3^ (*corresponding)

1. University of Liverpool Institute of Infection and Global Health, Ronald Ross Building, 8 West Derby Street, Liverpool, L69 7BE,

2. Department of Infectious Diseases, Alder Hey Children’s NHS Foundation Trust, Eaton Road, Liverpool, L12 2AP,

3. Liverpool Health Partners, 1st Floor, Liverpool Science Park, 131 Mount Pleasant, Liverpool, L3 5TF

4. University of Liverpool Institute of Integrative Biology, Biosciences Building, Crown Street
Liverpool L69 7ZB

5. Department of Critical Care, Alder Hey Children’s NHS Foundation Trust, Eaton Road, Liverpool, L12 2AP

6. Medicine, Ethics, Society & History, University of Birmingham.

7. Clinical Research Division, Alder Hey Children’s NHS Foundation Trust.

8. Department of Infectious Diseases, Oxford University Hospital,, Children’s Hospital , John Radcliffe Hospital, Oxford OX3 9DU

**Corresponding author:**

Professor Enitan D Carrol

University of Liverpool Institute of Infection and Global Health, Ronald Ross Building, 8 West Derby Street, Liverpool, L69 7BE

[edcarrol@liverpool.ac.uk](mailto:edcarrol@liverpool.ac.uk)

**Supplementary Figure 1**


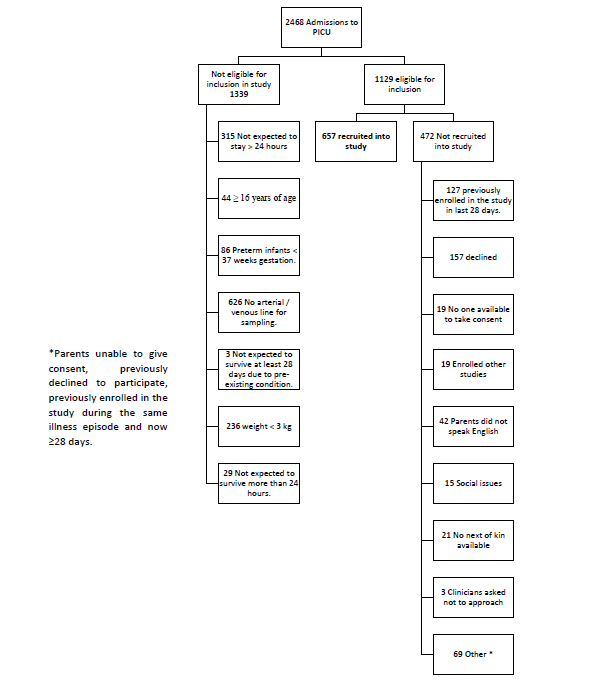


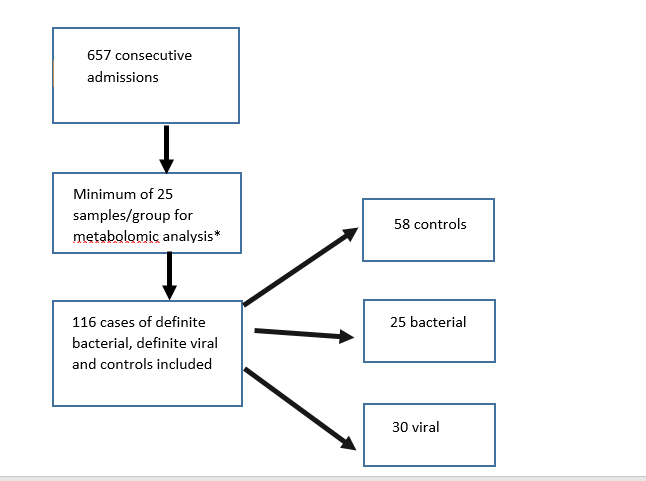


* sample size based upon 'clean' phenotype of defined criteria (see methods)

**Supplementary Figure 1:** Flow chart showing consecutive admissions to intensive care and selection of cases for metabolomics analysis

**Supplementary Figure 2.**

**
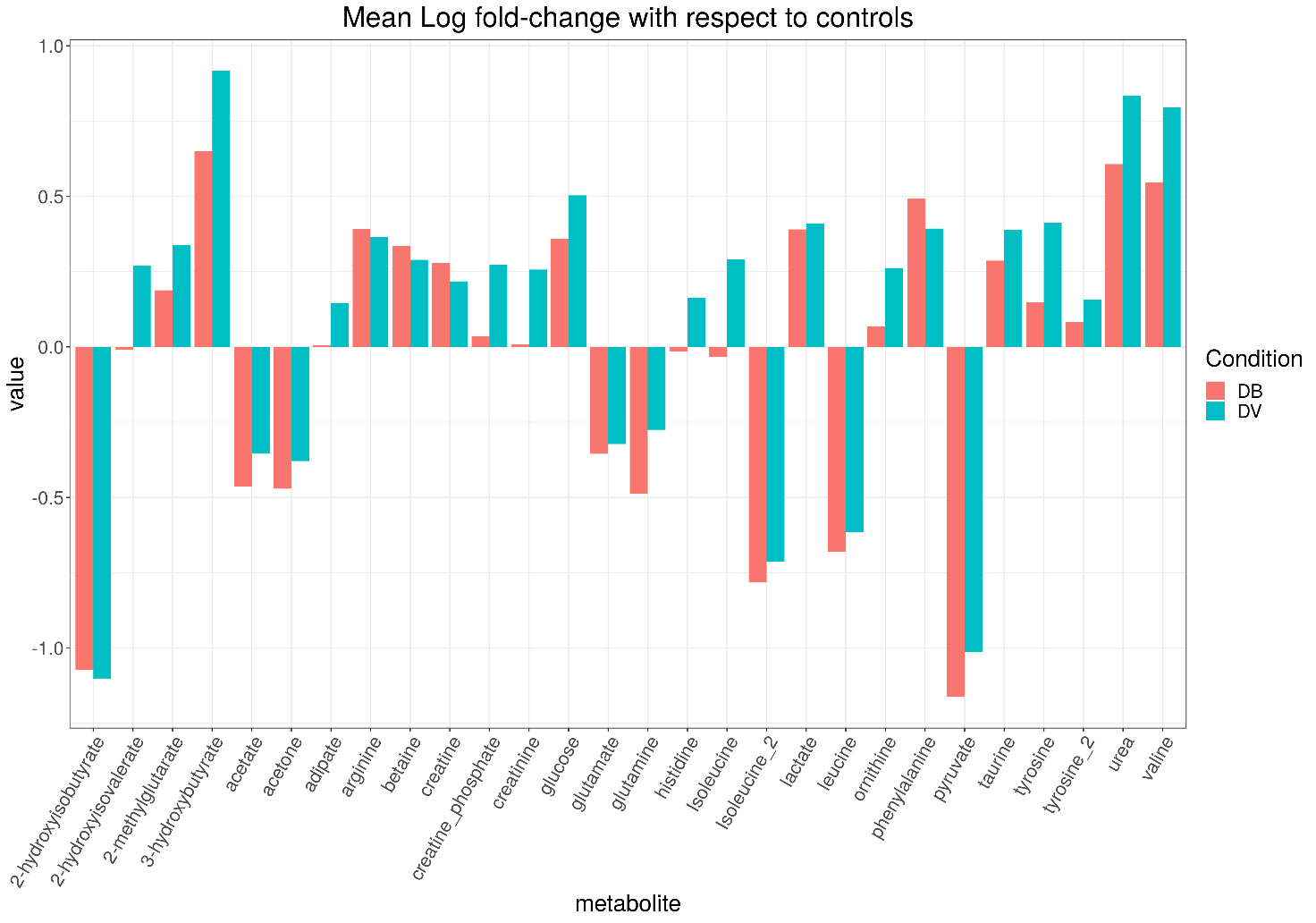
**

**Supplementary Figure 2.** Fold Change analysis (log2 scale) of DB (red) or DV (blue) metabolite levels compared to C group from aggregated list of metabolites contributing to PLS-DA models shown in Figure 2.

**Supplementary Figure 3**.


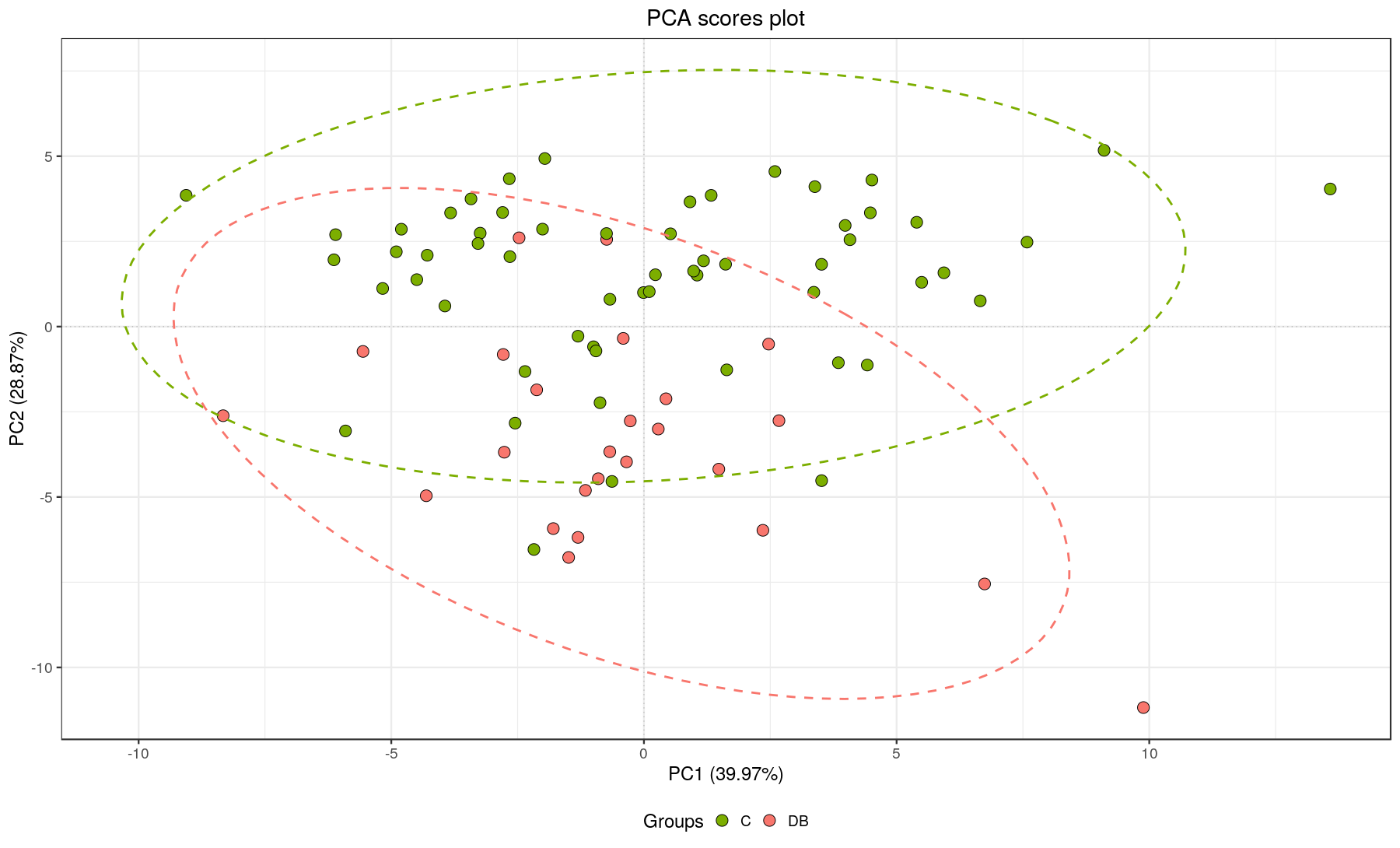


**Supplementary Figure 3**. PCA scores plot comparing C-DB samples. C-controls, DB-definite bacterial infection.

**Supplementary Figure 4**.


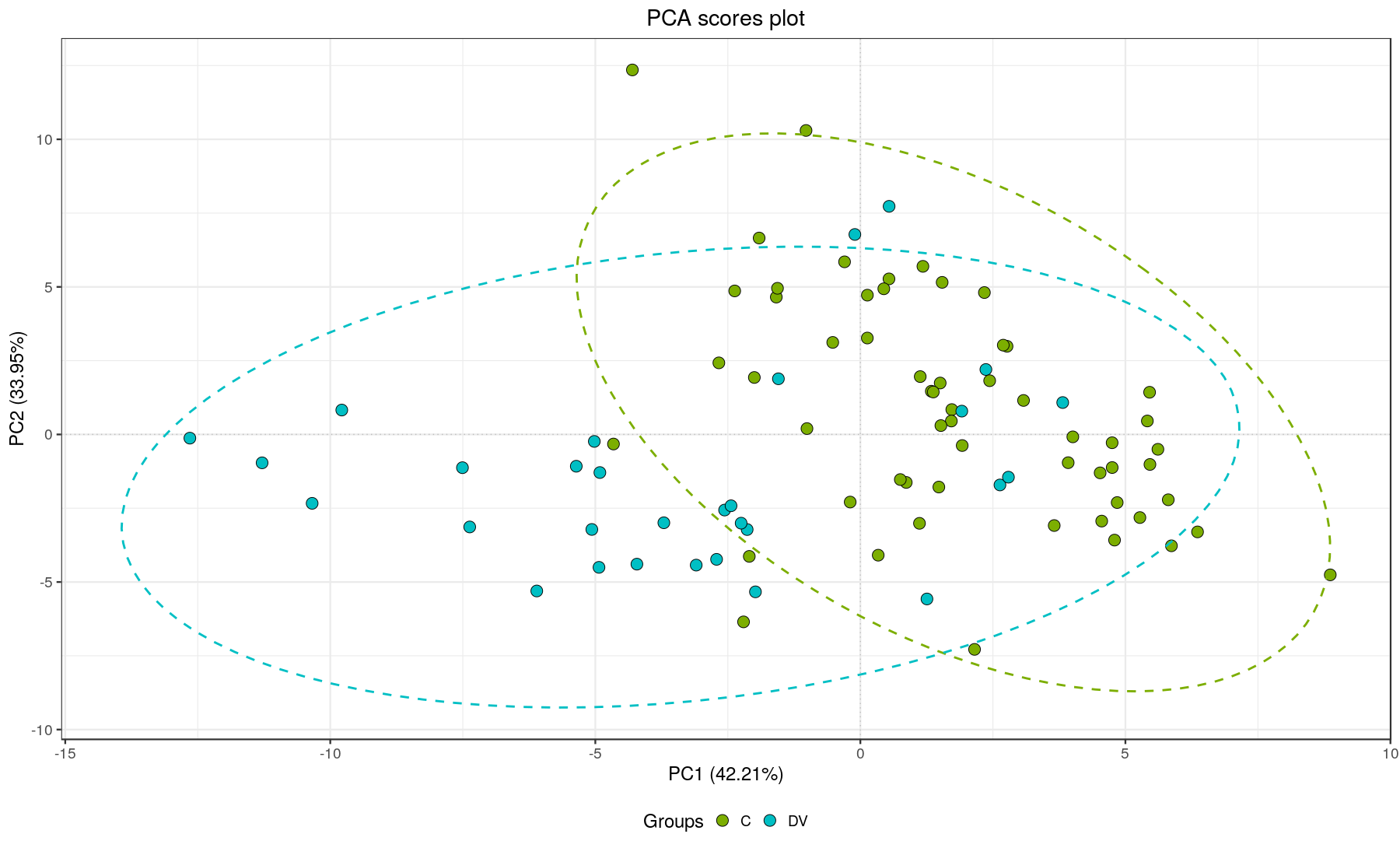


**Supplementary Figure 4**. PCA scores plot comparing C-DV samples. C-controls, DV-definite viral infection.

**Supplementary Figure 5**.

A)


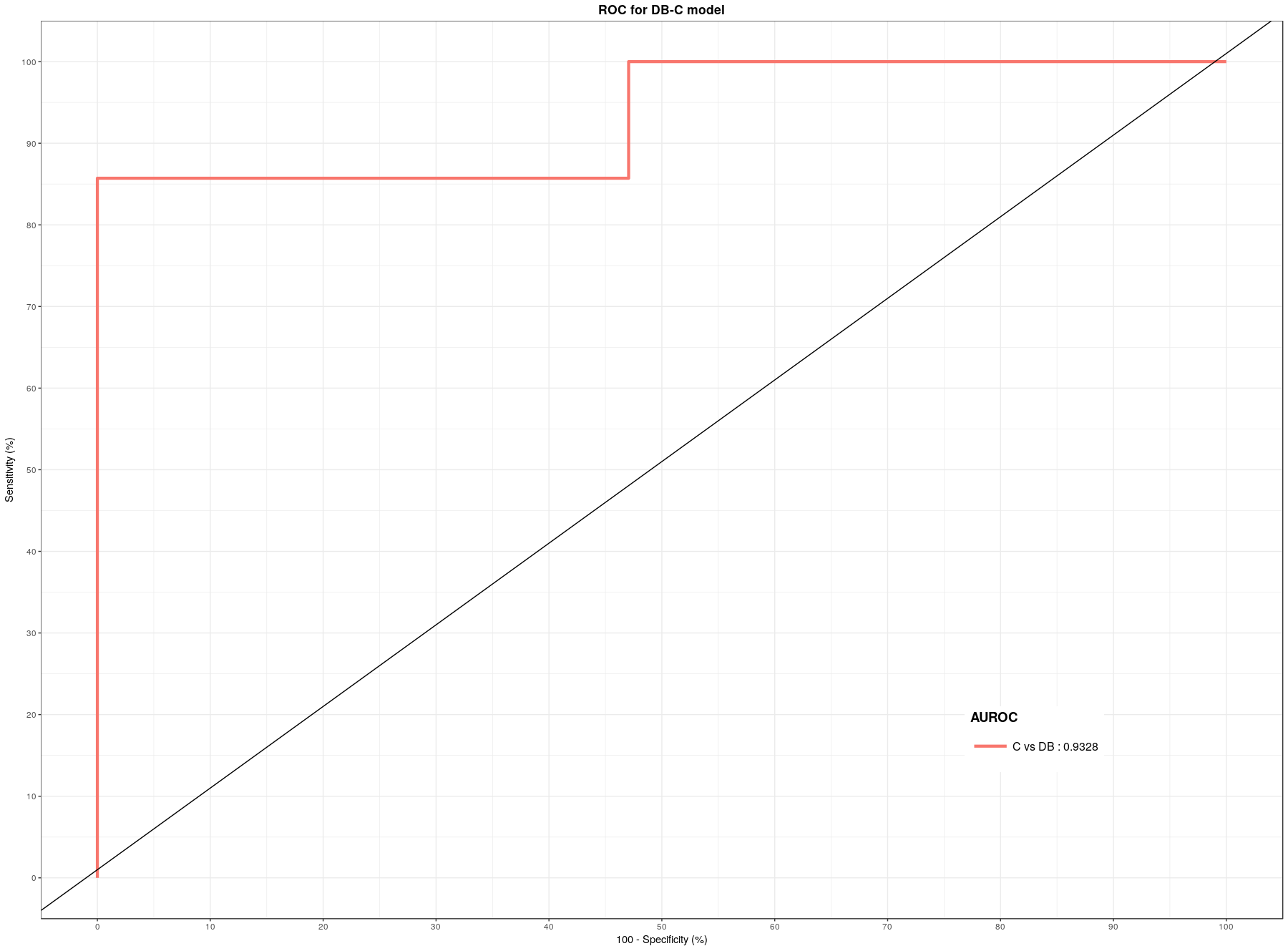


B)


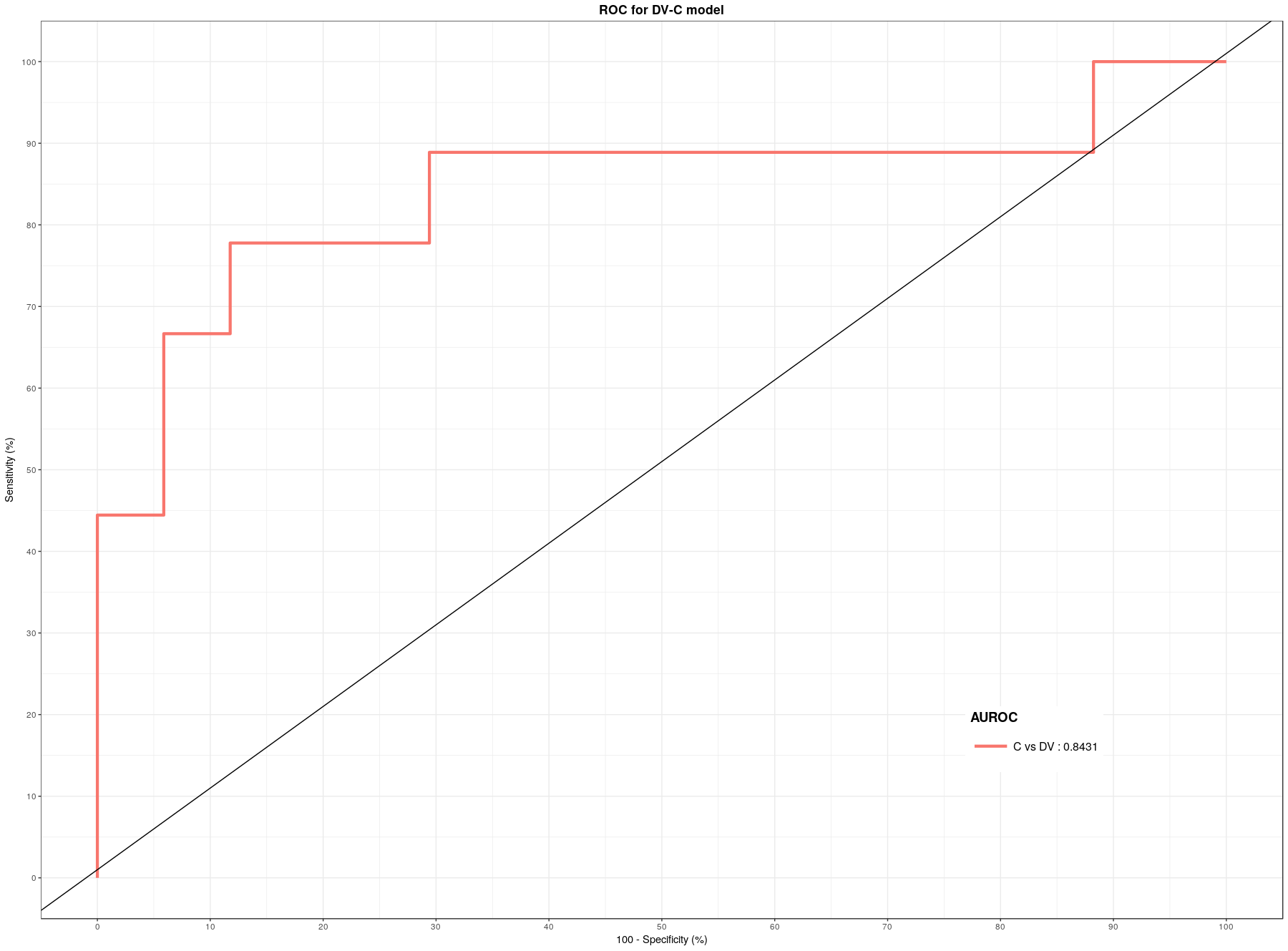


**Supplementary Figure 5**. A) PLSDA ROC curve for model comparing C-DB samples. C-controls, DB-Definite bacterial infection B) PLSDA ROC curve for model comparing C-DV samples. C-controls, DV-Definite viral infection.

**Supplementary Figure 6.**


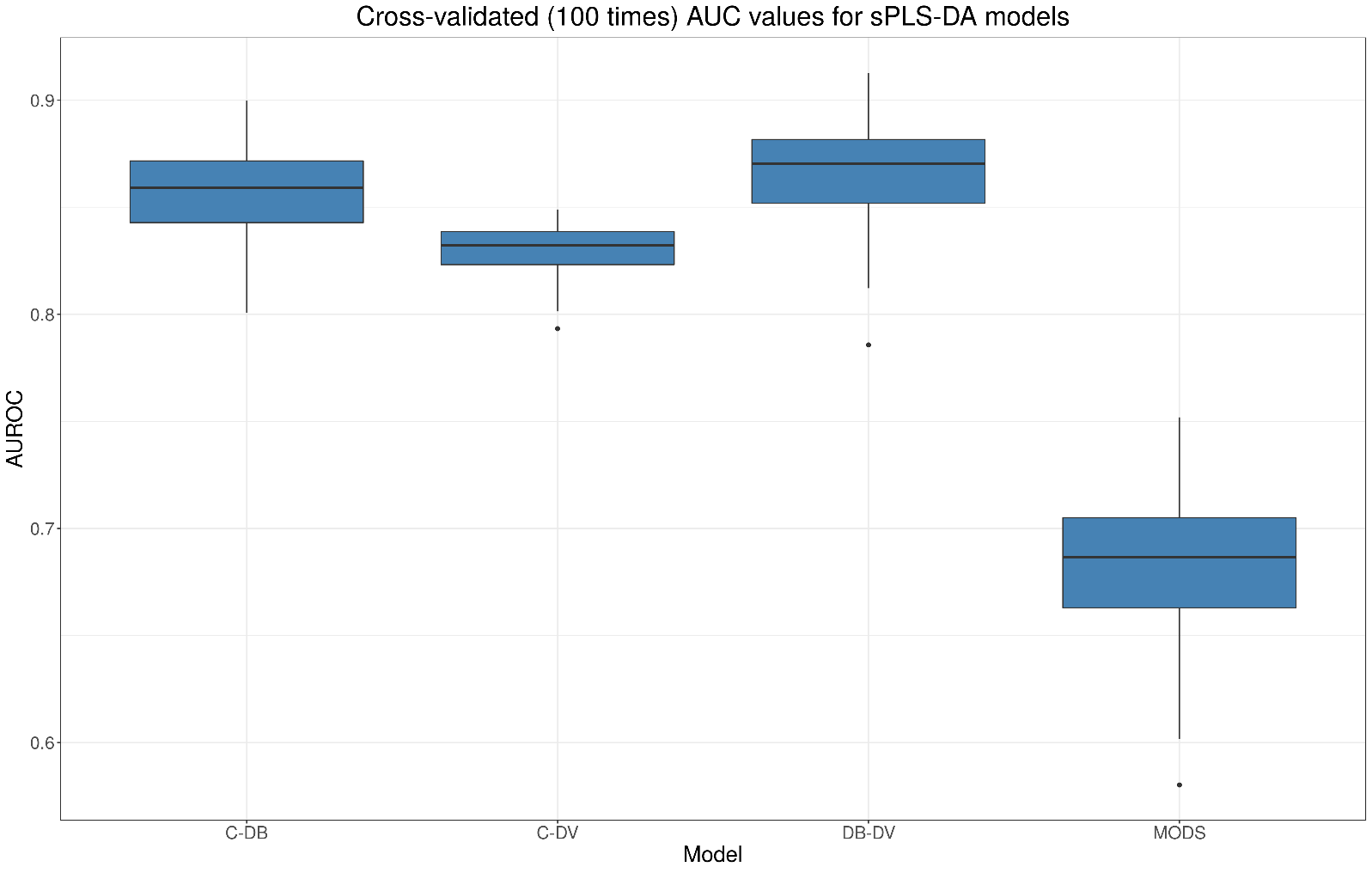


**Supplementary Figure 6.**. AUC values acquired from cross-validation of all sPLSDA models over 100 iterations each.

**Supplementary Figure 7.**


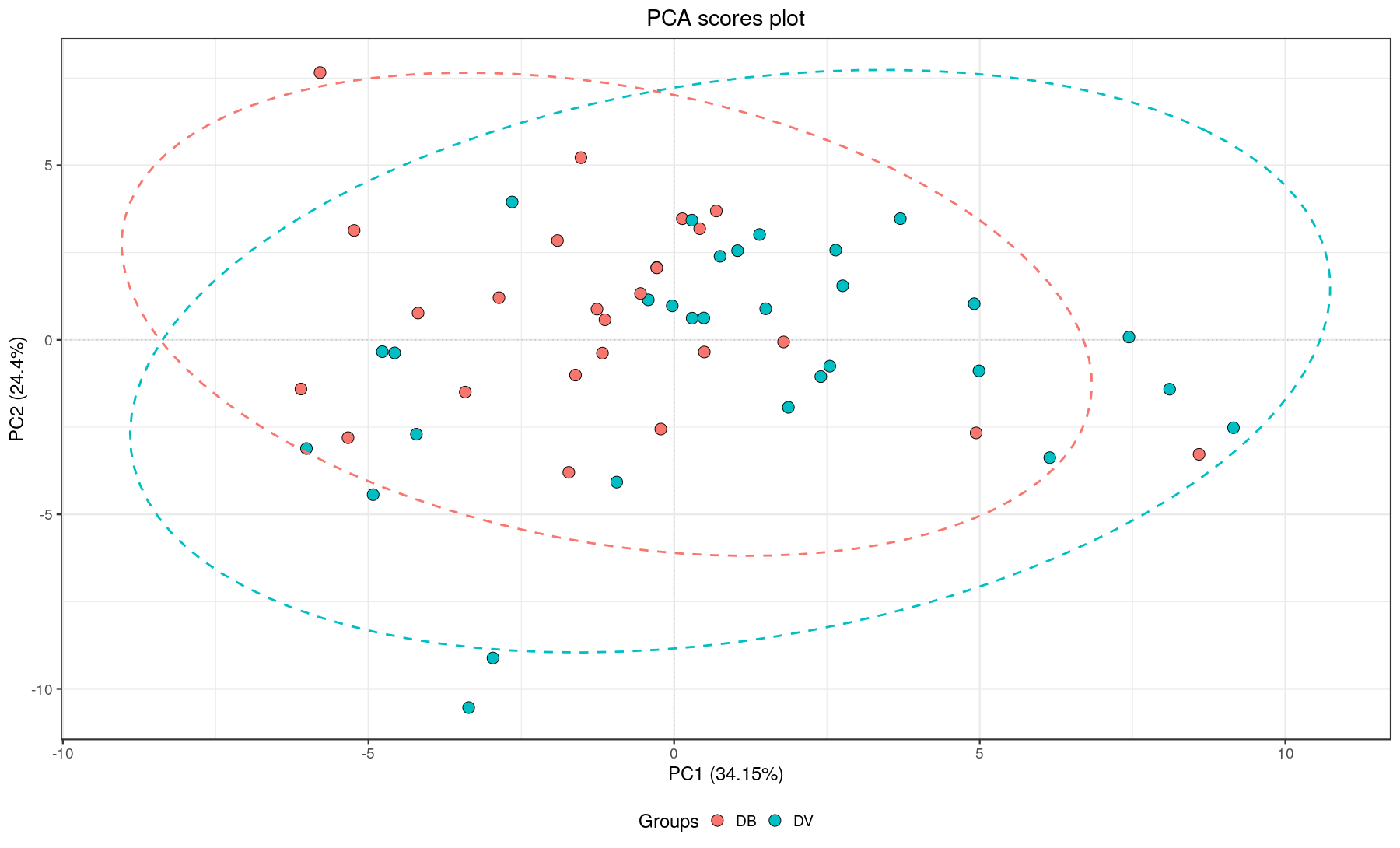


**Supplementary Figure 7**. PCA scores plot comparing DB-DV samples. DB-definite bacterial infection, DV-definite viral infection.

**Supplementary Figure 8**.


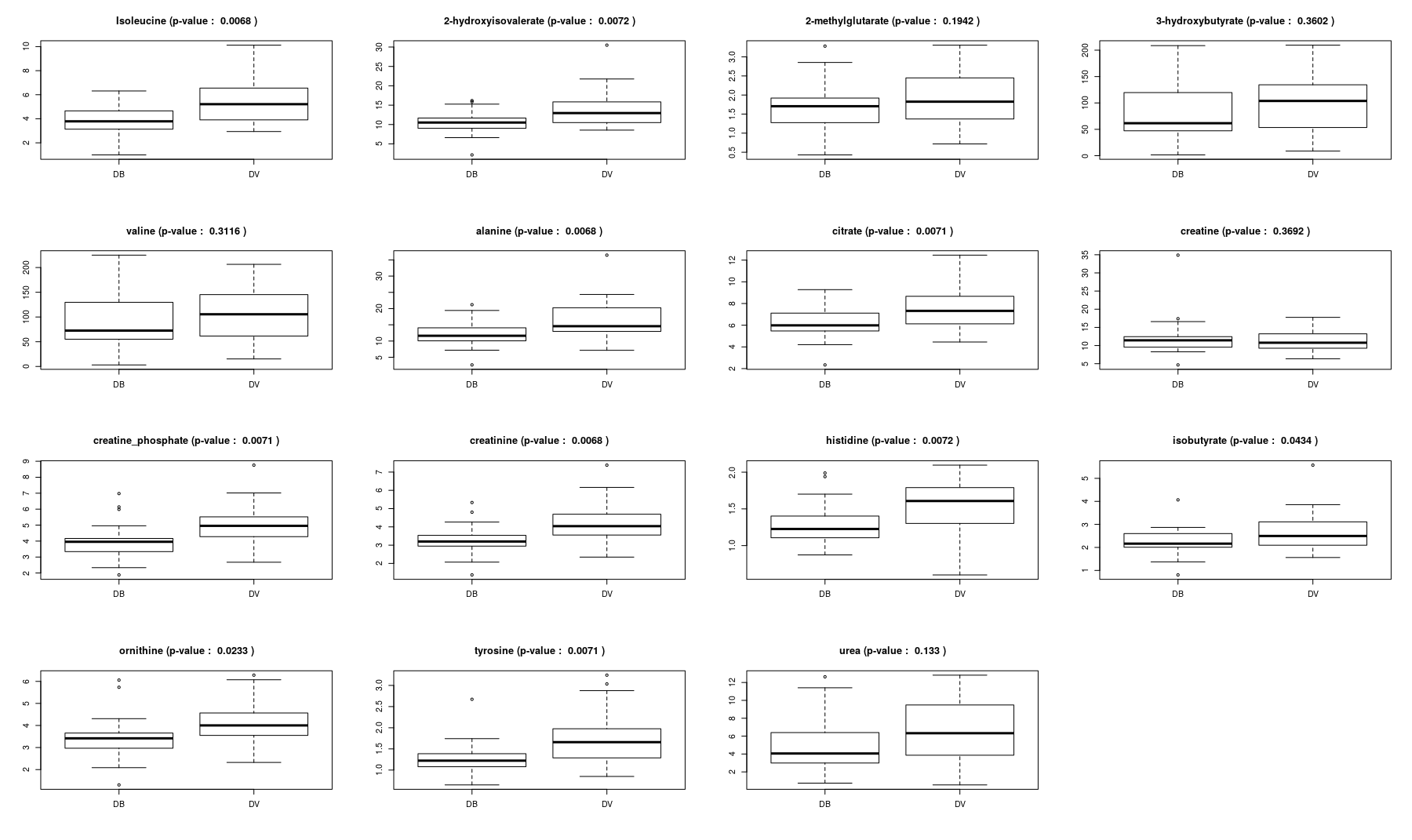


**Supplementary Figure 8**. Boxplots of most contributing metabolites in the PLS-DA model of bacterial vs viral infection.

**Supplementary Figure 9.**


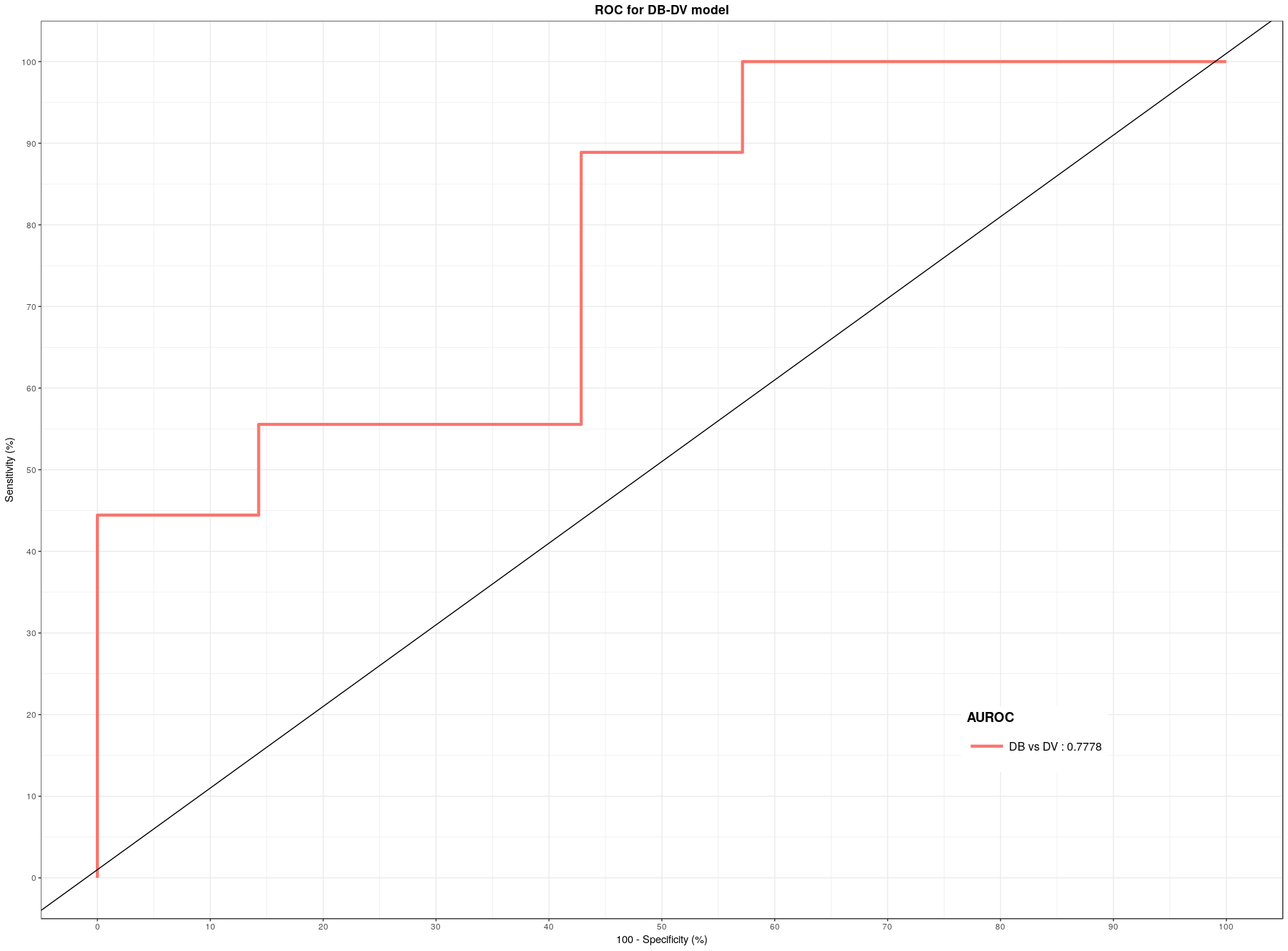


**Supplementary Figure 9**. PLSDA ROC curve for model comparing DB-DV samples. DB-definite bacterial infection, DV-Definite viral infection.

**Supplementary Figure 10**
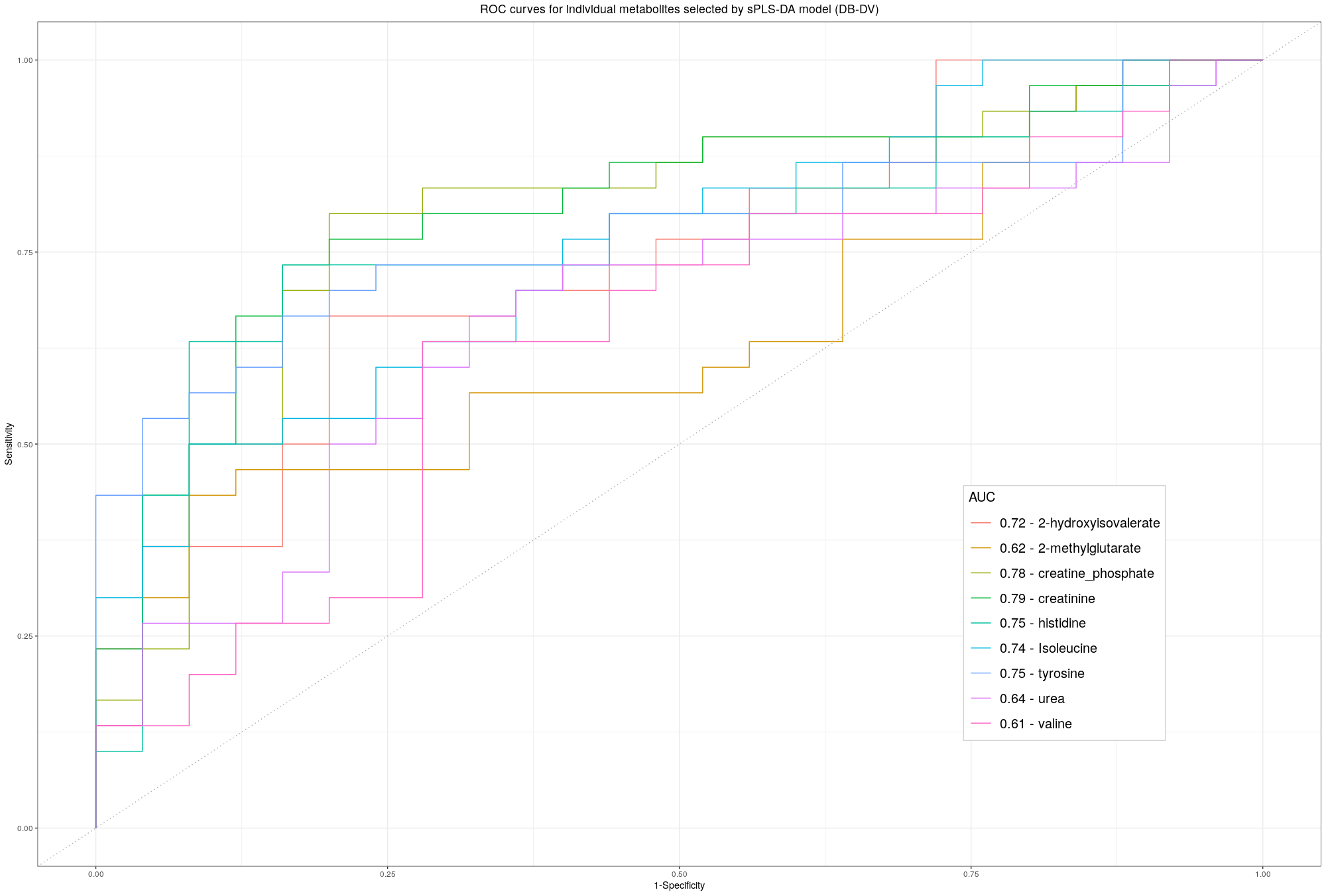


**Supplementary Figure 10**. ROC curves for individual metabolites selected by sPLS-DA model comparing patients with bacterial infection (DB) to patients with viral infection (DV).

**Supplementary Figure 11**


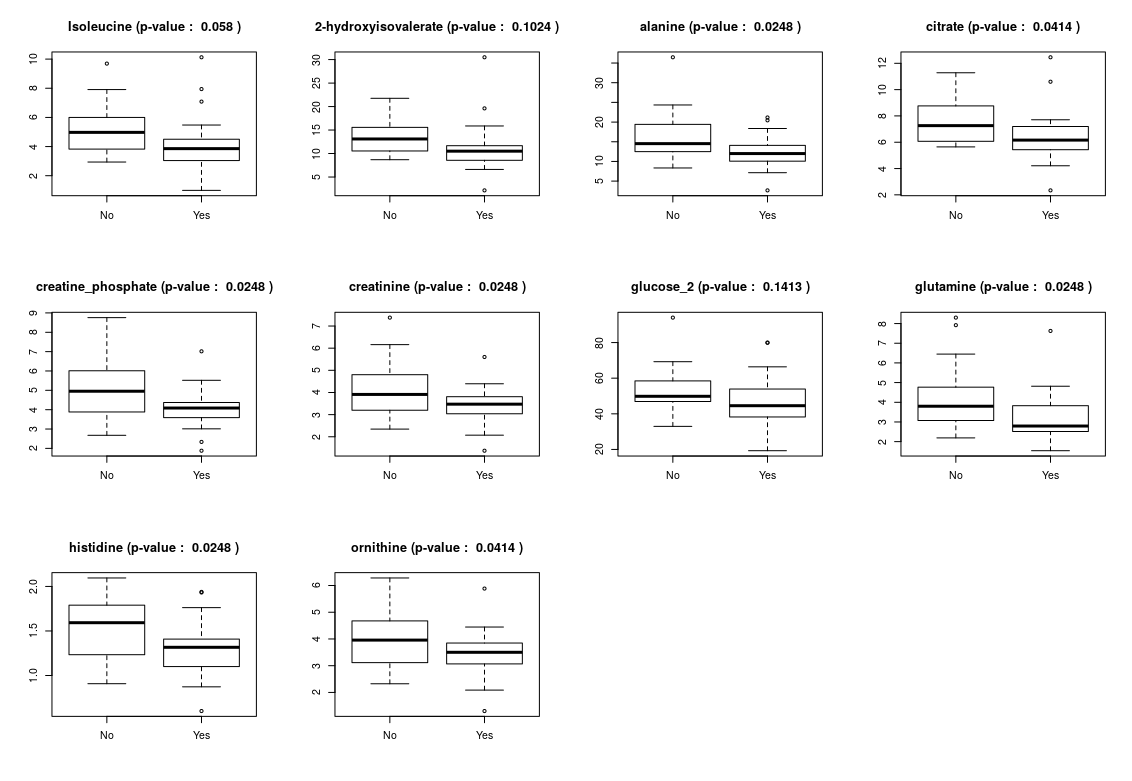


**Supplementary Figure 11** Boxplots of most contributing metabolites in the PLS-DA model of organ-dysfunction

**Supplementary Figure 12**

**
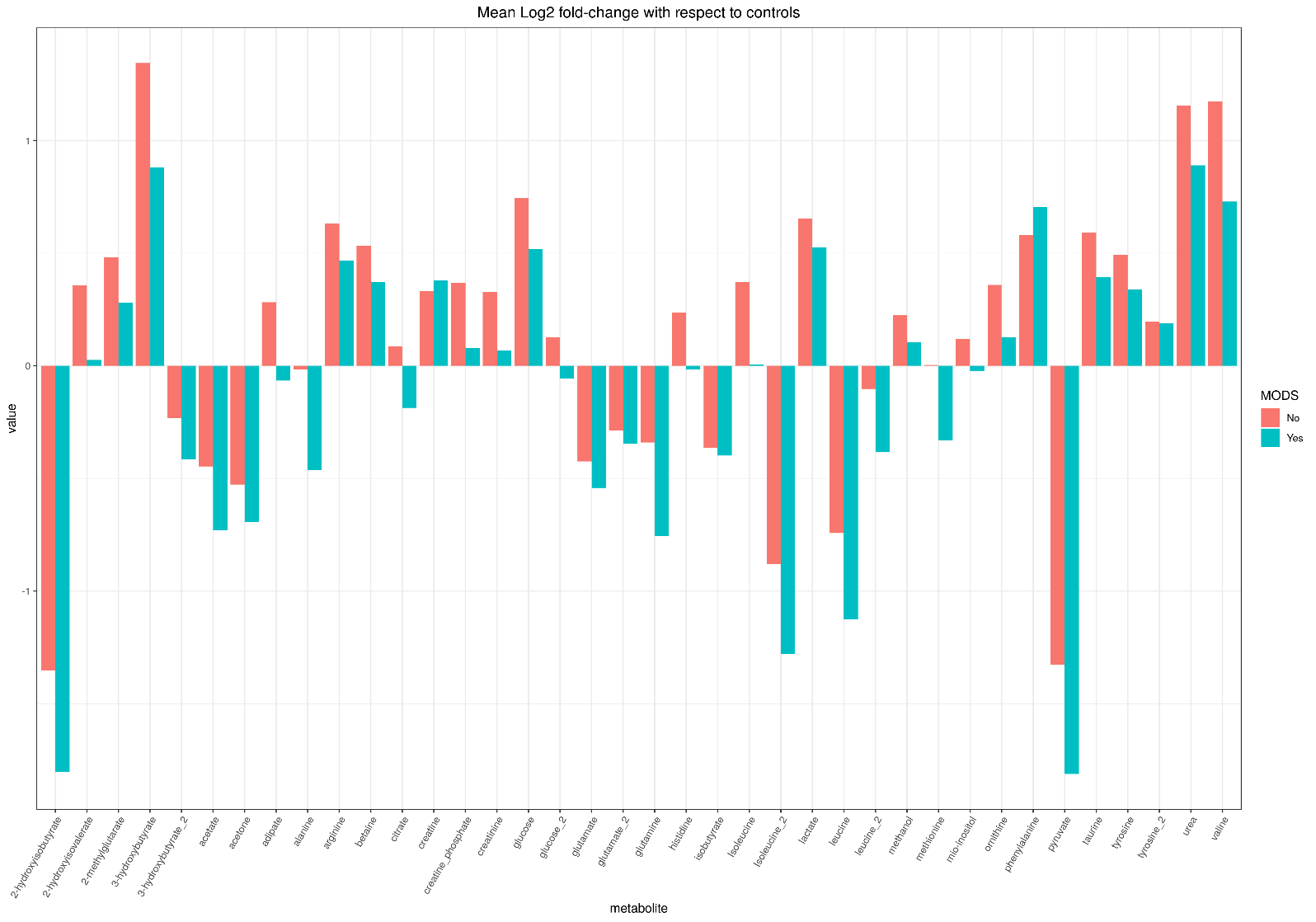
**

**Supplementary Figure 12.** Fold Change analysis (log2 scale) of MOD positive (red) and MOD negative (blue) metabolite levels compared to control.

**Supplementary Figure 13**

**
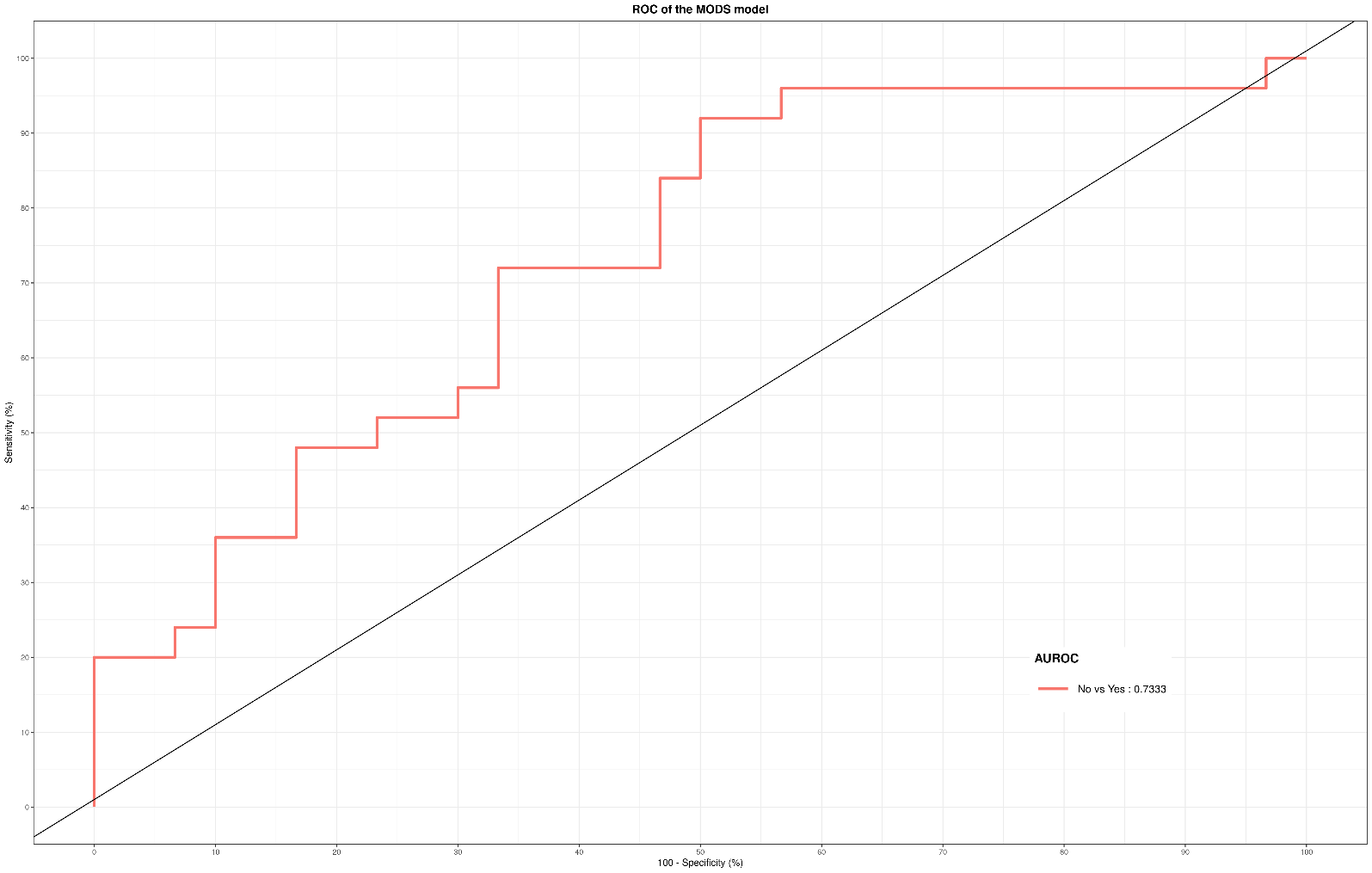
**

**Supplementary Figure 13.** ROC Curve for PLS-DA model discriminating between MOD positive and MOD negative patients with infection. AUC=0.73.

**Supplementary Figure 14
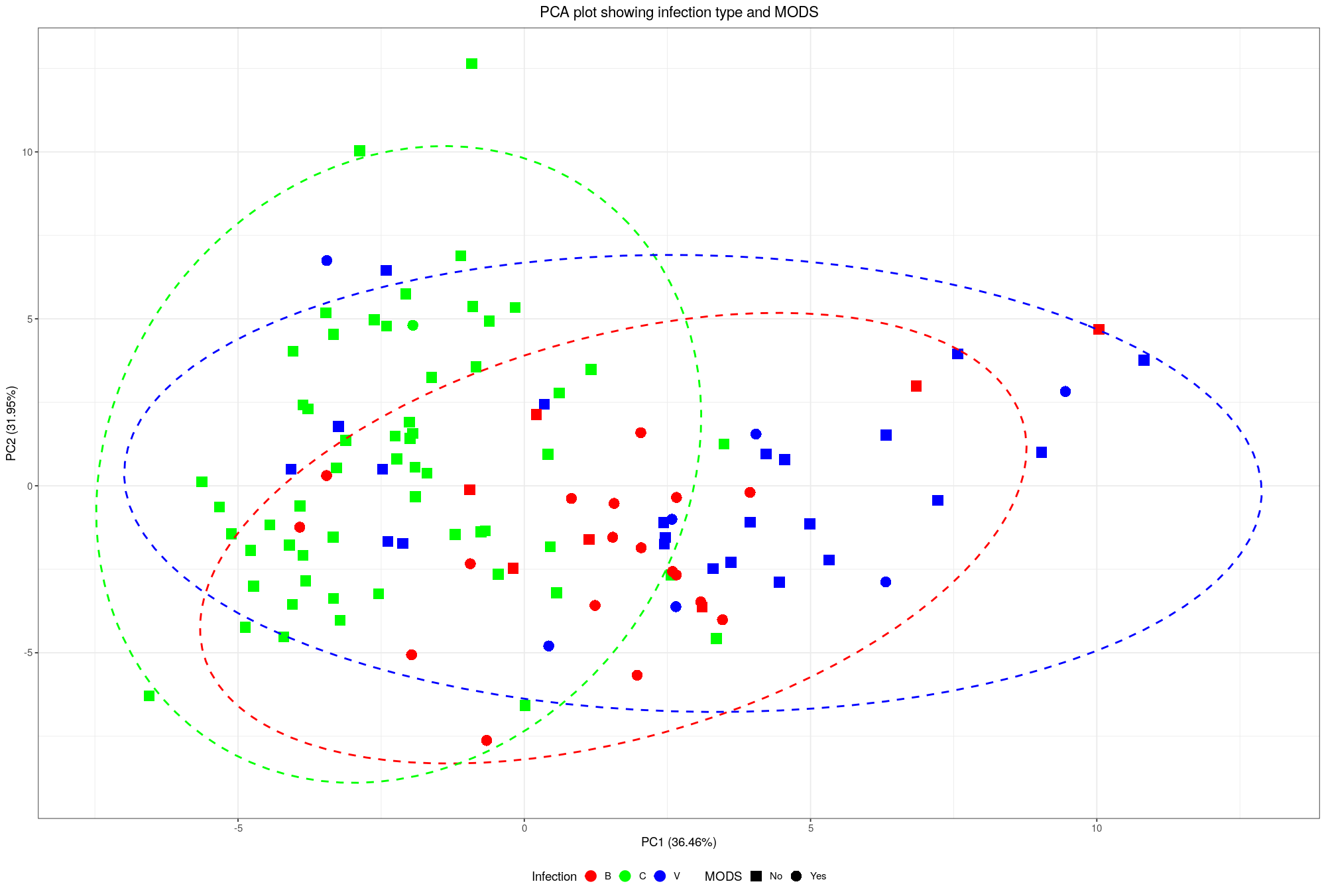
**

**Supplementary Figure 14.** PCA of the samples showing infection groups as well as MOD status.

**Supplementary Figure 15**

**
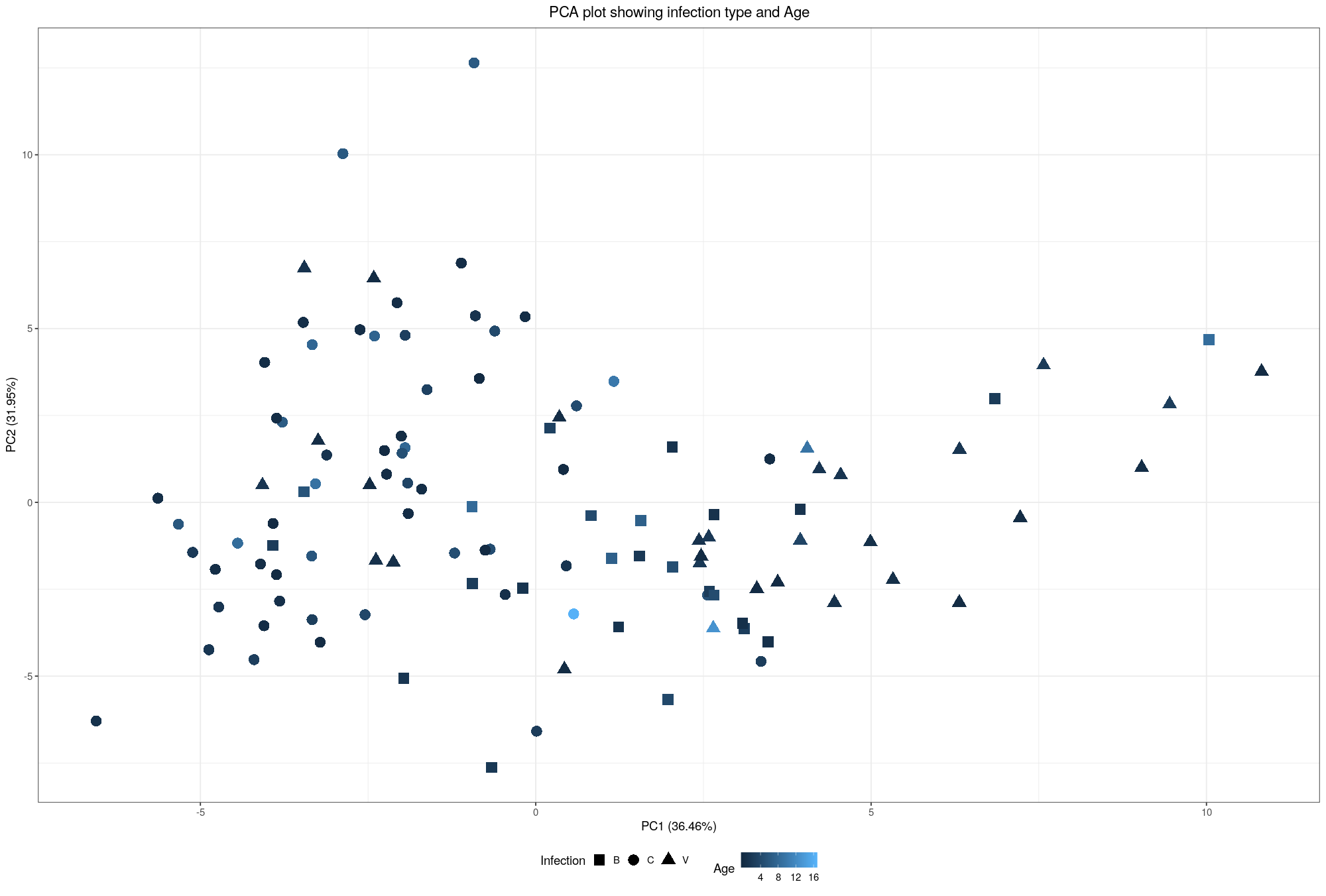
**

**Supplementary Figure 15**. PCA of the samples showing infection groups as well as patient age on admission.

**Supplementary Table 1.** Correlations between selected metabolites (DB-DV) and biochemical test.

|  | Isoleucine | 2-hydroxy-isovalerate | tyrosine | creatinine | valine | Creatine phosphate | histidine | 2-methyl-glutarate | urea |
| --- | --- | --- | --- | --- | --- | --- | --- | --- | --- |
| HB | -0.00869 | -0.06154 | 0.02038 | 0.135822 | 0.08298 | 0.080556 | -0.03083 | 0.202368 | 0.050427 |
| Lymph | -0.09163 | -0.19351 | -0.11908 | -0.09662 | 0.116676 | -0.07652 | 0.072188 | -0.04363 | 0.10225 |
| WBC | -0.02737 | -0.09319 | -0.14116 | -0.30254 | 0.187645 | -0.20213 | -0.25456 | 0.061976 | 0.156204 |
| Neutrophils | -0.02273 | -0.06209 | -0.13266 | -0.30579 | 0.136324 | -0.2103 | -0.29357 | 0.043911 | 0.106072 |
| Platelet | 0.394957 | 0.310604 | 0.384594 | 0.272849 | 0.305408 | 0.322884 | 0.089573 | 0.452041 | 0.383445 |
| CRP | -0.24977 | -0.28284 | -0.25824 | -0.35184 | 0.106829 | -0.23492 | -0.44085 | -0.09117 | 0.007075 |
| ALT | -0.10984 | 0.014945 | 0.048227 | 0.050644 | -0.11716 | 0.050174 | 0.156857 | 0.097758 | -0.0306 |
| AST | -0.24372 | -0.17204 | -0.11626 | -0.10724 | -0.30356 | -0.1393 | 0.061239 | -0.20736 | -0.26264 |
| Creatinine | -0.40705 | -0.39036 | -0.44179 | -0.3215 | -0.28888 | -0.31793 | -0.37881 | -0.20811 | -0.31171 |
| Urea | -0.46705 | -0.40642 | -0.4059 | -0.33682 | -0.34782 | -0.38861 | -0.22317 | -0.26639 | -0.37941 |
| Fib | -0.16215 | -0.19666 | -0.12691 | -0.09355 | 0.199648 | -0.13543 | 0.073441 | -0.01718 | 0.15284 |
| Bilirubin | -0.39912 | -0.29373 | -0.0608 | 0.024122 | -0.2952 | -0.05895 | 0.039688 | -0.09536 | -0.26952 |
| APPT | -0.32317 | -0.30149 | -0.33575 | -0.17093 | -0.20426 | -0.24089 | -0.1499 | -0.28972 | -0.23721 |
| INR | -0.32937 | -0.34481 | -0.30895 | -0.14933 | -0.05273 | -0.22666 | 0.027426 | -0.24632 | -0.10272 |
| PT | -0.3864 | -0.38779 | -0.37214 | -0.15711 | -0.18728 | -0.24223 | -0.00216 | -0.34548 | -0.23775 |
